# Detecting Nonvolatile Life- and Nonlife-Derived Organics in a Carbonaceous Chondrite Analogue with a New Multiplex Immunoassay and Its Relevance for Planetary Exploration

**DOI:** 10.1089/ast.2017.1747

**Published:** 2018-08-01

**Authors:** Mercedes Moreno-Paz, Ana Gómez-Cifuentes, Marta Ruiz-Bermejo, Oliver Hofstetter, Ángel Maquieira, Juan M. Manchado, Sergi Morais, Mark A. Sephton, Reinhard Niessner, Dietmar Knopp, Victor Parro

**Affiliations:** ^1^ Department of Molecular Evolution, Centro de Astrobiología (INTA-CSIC), Madrid, Spain.; ^2^ Department of Chemistry and Biochemistry, Northern Illinois University, DeKalb, Illinois.; ^3^ Department of Chemistry, Instituto Universitario de Reconocimiento Molecular y Desarrollo Tecnológico, Universidad Politécnica de Valencia, Valencia, Spain.; ^4^ Department of Earth Science and Engineering, Imperial College London, London, United Kingdom.; ^5^ Department Chemie, Technische Universität München, Munich, Germany.

**Keywords:** Planetary exploration, Molecular biomarkers, D- and L-aromatic amino acids, Life detection, Multiplex inhibitory/competitive immunoassay, Kerogen type IV

## Abstract

Potential martian molecular targets include those supplied by meteoritic carbonaceous chondrites such as amino acids and polycyclic aromatic hydrocarbons and true biomarkers stemming from any hypothetical martian biota (organic architectures that can be directly related to once-living organisms). Heat extraction and pyrolysis-based methods currently used in planetary exploration are highly aggressive and very often modify the target molecules, making their identification a cumbersome task. We have developed and validated a mild, nondestructive, multiplex inhibitory microarray immunoassay and demonstrated its implementation in the SOLID (Signs of Life Detector) instrument for simultaneous detection of several nonvolatile life- and nonlife-derived organic molecules relevant in planetary exploration and environmental monitoring. By utilizing a set of highly specific antibodies that recognize D- or L-aromatic amino acids (Phe, Tyr, Trp), benzo[a]pyrene (B[a]P), pentachlorophenol, and sulfone-containing aromatic compounds, respectively, the assay was validated in the SOLID instrument for the analysis of carbon-rich samples used as analogues of the organic material in carbonaceous chondrites or even Mars samples. Most of the antibodies enabled sensitivities at the 1–10 ppb level and some even at the part-per-trillion level. The multiplex immunoassay allowed the detection of B[a]P as well as aromatic sulfones in a water/methanol extract of an Early Cretaceous lignite sample (ca. 140 Ma) representing type IV kerogen. No L- or D-aromatic amino acids were detected, reflecting the advanced diagenetic stage and the fossil nature of the sample. The results demonstrate the ability of the liquid extraction by ultrasonication and the versatility of the multiplex inhibitory immunoassays in the SOLID instrument to discriminate between organic matter derived from life and nonlife processes, an essential step toward life detection outside Earth.

## 1. Introduction

In the field of Exobiology/Astrobiology, the detection of molecular evidence of life has been a challenging goal since the beginning of space exploration. Molecular targets for life detection encompass a wide variety of molecules; many of them are low-molecular-weight compounds, which are probably present only at very low concentration and, thus, difficult to detect and identify by *in situ* analytical devices (Parnell *et al.*, 2007). The rich organic chemistry of meteorites, containing molecules such as amino acids and polycyclic aromatic hydrocarbons (PAHs) (Ehrenfreund *et al.*, 2001; Burton *et al.*, 2012), fed the surface of Mars with organic matter billions of years ago (Grotzinger *et al.*, 2010). Although the Viking missions in the mid-1970s failed to detect any trace of organic compounds (Biemann, 1979), the Sample Analysis at Mars (SAM) instrument of NASA's Mars Science Laboratory (MSL) has recently detected and identified several chlorinated organic compounds in sedimentary rocks at Gale Crater, among them from 150 to 300 ppb of chlorobenzene, the most complex molecule identified so far on Mars (Freissinet *et al.*, 2015). This aromatic compound has been identified by direct thermal evolved gas analysis, probably as of PAHs or PAH-rich refractory material as it was first suggested by Ming *et al.* (2014).

The harsh conditions on the surface and near subsurface of Mars, such as UV and penetrating ionizing radiation, and the presence of chlorine species and other chemicals can oxidize, degrade, or partially damage organic matter (Rix *et al.*, 2011; Lewis *et al.*, 2015) before analysis. The sample preparation methods used on Mars by the *in situ* analytical techniques up to now are mainly based on thermal and pyrolytic extraction of volatile compounds. High temperatures can produce additional degradation of the already irradiated and damaged organic compounds as a consequence of the heat alone, or in combination with the strong oxidative effect of perchlorate at elevated temperatures (Navarro-González *et al.*, 2010). Two effects are expected, a lower concentration of organic matter in the martian samples by long-term harsh conditions, and a loss of structural and taphonomical information of the original molecules after the analysis. Therefore, future missions require sensitive measurement capabilities to detect the remaining undegraded compounds, with gentler methods that help to minimize any further degradation while maintaining the original (biological or not) molecular information.

Gentle, low-temperature extraction methods, such as ultrasonication in liquid solvents, and nondestructive analytical methods, such as immunoassays, can respond to those requirements for obtaining molecular information in planetary exploration (Parro *et al.*, 2005, 2008; Parro *et al.*, 2011a,b; Sims *et al.*, 2012). Moreover, such methods promise to address the main challenges of space exploration, namely, the development of instrumentation suitable for expeditions within our solar system, particularly the search for molecular signs of life on Mars. In recent years, we have proposed the antibody microarray-based SOLID (Signs of Life Detector) instrument, which allows wet and low-temperature analysis of both solid and liquid samples (Parro *et al.*, 2005, 2008; Parro *et al.*, 2011a, 2011b). SOLID 3.0 (Parro *et al.*, 2011b) has two functional units: The Sample Preparation Unit (SPU), which extracts the organic matter into a liquid buffer by ultrasonication, and the Sample Analysis Unit (SAU), which analyzes this extract by highly specific immunological assays. The system has been used successfully in several field campaigns and environments such as the Antarctica and the Atacama Desert, which are both considered good Mars analogues (Parro *et al.*, 2011a; Blanco *et al.*, 2012).

In the last two decades, microarray technology has played an invaluable role in the investigation of biological systems and, thus, the advancement of fields such as biomedicine and ecology. Ever since MacBeath *et al.* (1999) developed the first microarray for the analysis of small molecules based on protein/ligand interactions, this approach has been extended to multiplex inhibitory microarray immunoassays (MIMI) and has found numerous applications, including the detection of pollutants and toxins (Szkoda *et al.*, 2014; Carter *et al.*, 2016), drugs and antibiotics (Noguera *et al.*, 2002; Gonzalez-Martinez *et al.*, 2003; Tamarit-López *et al.*, 2010; Wang *et al.*, 2013), polysaccharides (Pickering *et al.*, 2002), stereoisomers of amino- and hydroxy acids (Kassa *et al.*, 2011), as well as naphthalene and benzo[a]pyrene (B[a]P) (Matschulat *et al.*, 2005), including for planetary exploration purposes (Fernández-Calvo *et al.*, 2006; Rix *et al.*, 2011). A major advantage of MIMI is the capability to analyze multiple organic compounds of a wide range of molecular sizes, from amino acids to whole cells and spores (Fernández-Calvo *et al.*, 2006) in a single assay, even if they are contained in soil or complex samples (Rodriguez-Mozaz *et al.*, 2006a,b).

Demonstration of the ability of analytical instruments to discriminate between organic matter derived from life and nonlife processes is an essential step toward life detection outside Earth. Prebiotic chemistry and highly altered and degraded biological chemistry may overlap by sharing compounds (amino acids, nucleobases, PAHs) whose origin is difficult to decipher unless fine structural details and/or enantiomeric and isotopic ratios are determined. The comparison of biologically altered Earth samples that are millions of years old with samples enriched in prebiotic chemicals such as the carbonaceous chondrites is relevant to identify true biomarkers at the border of prebiotic and biotic chemistry.

Carbonaceous chondrites are a rare, but important, class of meteorites that contain a high percentage of water and organic compounds (Pizzarello and Shock, 2010). However, their scarce availability has led scientists to use terrestrial materials that possess properties analogous to carbonaceous chondrites and meteorites, such as kerogen-containing samples, for the development of analytical methods. For example, Matthewman *et al.* (2013) compared several organic matter types with the macromolecular material-dominated organic matter present in the Murchison Meteorite, and concluded that the aromatic-dominated organic matter types, namely, type IV oxidized “lignites” such as those from Early Cretaceous fluvial sands and type IV organic matter such as that present in Late Jurassic paleosols, are plausible analogues for carbonaceous chondrite organic matter and potential organic matter-containing martian sediments.

The term kerogen refers to the insoluble organic matter present in sedimentary rocks that is sometimes highly enriched in PAHs and sulfur bearing heterocycles, and not extractable with organic solvents (Rullkötter and Michaelis, 1990). In terms of the relative proportions of different organic materials contained, as a function of their diagenetic maturation (the effect of high pressure and temperature), kerogens are classified into four types (I–IV) (Tissot *et al.*, 1974; Larter and Senftle, 1985). Type IV kerogen contains material produced by reworking and oxidation of organic matter, and is characterized by its extremely low H and O contents. Matthewman *et al.* (2013) suggested that kerogen type IV samples can be considered analogues of carbonaceous chondrites for developing and testing new methods to distinguish potential biomarkers from probiotic compounds in planetary exploration.

Here, we describe the development of an MIMI for the detection of small molecules, and its validation with type IV organic matter-containing samples that bear superficial organic chemical similarities to that found in carbonaceous chondrites. Antibodies against a variety of different organic compounds, including aromatic amino acids, drugs, PAHs, and peptides, were tested, and the assay was implemented in the SOLID instrument for planetary exploration.

## 2. Materials and Methods

### 2.1. Analytes and antibodies

**FIG. 1.**
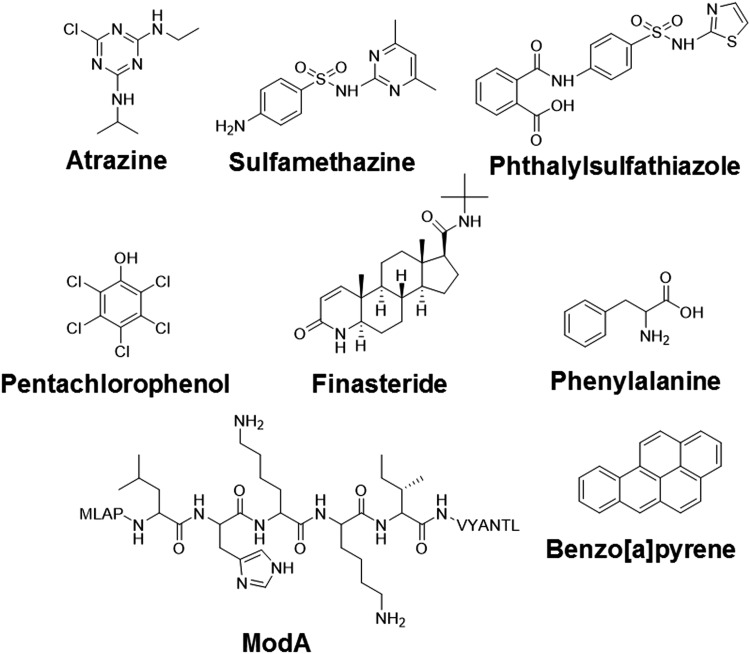
Molecular structures of the organic compounds used in this work as haptens or free analytes. All analytes were conjugated to different proteins (BSA, KLH, or OVA) for printing on epoxy-activated glass slides. BSA, bovine serum albumin; KLH, keyhole limpet hemocyanin; OVA, ovalbumin.

A total of 10 different antibodies (Table 1) were used, namely, two stereoselective anti-amino acid antibodies (anti-L-AA 18.3 and anti-D-AA) that recognize either L- or D- aromatic amino acids; two specific antibodies that bind to PAH B[a]P (anti-B[a]P); an antibody against a 15 aa peptide (anti-ModA peptide); and five antibodies against synthetic aromatic compounds (anti-atrazine, anti-finasteride, anti-phthalylsulfathiazole, anti-pentachlorophenol, and anti-sulfamethazine), all of which are used as herbicides or drugs (Fig. 1). Details on the production and characterization of the antibodies can be found elsewhere (Table 1).

**Table 1.** Analyte and Antibody Pairs Used in This Study

*Antibody*	*Analyte conjugate*	*Competitor/inhibitor*	*LOD (ppb)*	*IC_50_ (ppb)*^a^	*IC_100_ (ppm)*^a^	*Reference*
Anti-atrazine	Atrazine-OVA-2d	Atrazine (ATZ)	0.025	1	10	Tamarit-López *et al.* (2010)
Anti-sulfamethazine	Sulfamethazine-OVA-S6	Sulfamethazine (SMZ)	10	100	10	Pastor-Navarro *et al.* (2007)
Anti-phthalylsulfathiazole	Phthalylsulfathiazole-OVA-S8	Phthalylsulfathiazole (PSTZ)	10	300	100	Pastor-Navarro *et al.* (2007)
Anti-pentachlorophenol	Pentachlorophenol-OVA-PCP2	Pentachlorophenol (PCP)	3	10	1	Noguera *et al.* (2002)
Anti-finasteride	Finasteride-Hem-a1	Finasteride (FINA)	0.001	0.025	0.1	Brun *et al.* (2010)
Anti-L-AA 18.3 (mAb)	L-phenylalanine-BSA	L-Phe	500	2 × 10^4^	1000	Hofstetter *et al.* (2000)
Anti-D-AA (pAb)	D-phenylalanine-BSA	D-Phe	100	1 × 10^3^	10	Hofstetter *et al.* (1998)
Anti-ModA (pAb)	ModA-KLH	ModA	0.1	1	1	Fernández-Calvo *et al.* (2006)
Anti-B[a]P-4F11 (mAb)	(B[a]P-6-C3)-BSA	Benzo[a]pyrene (B[a]P)	0.001	0.1	10	Karsunke *et al.* (2011)
Anti-B[a]P-5G1 (mAb)	(B[a]P-6-C4)-BSA	Benzo[a]pyrene (B[a]P)	100	1.6 × 10^3^	10	Karsunke *et al.* (2011)

Multiplex inhibitory microarray immunoassays were performed as described in Section 2. The relative intensity values were corrected by their local background and by the negative control array (only buffer, without analytes).

^a^ Analyte concentration for 50% (IC_50_) or 100% (IC_100_) inhibition.

B[a]P = benzo[a]pyrene; BSA = bovine serum albumin; KLH = keyhole limpet hemocyanin; LOD = limit of detection; OVA, ovalbumin.

**FIG. 2.**
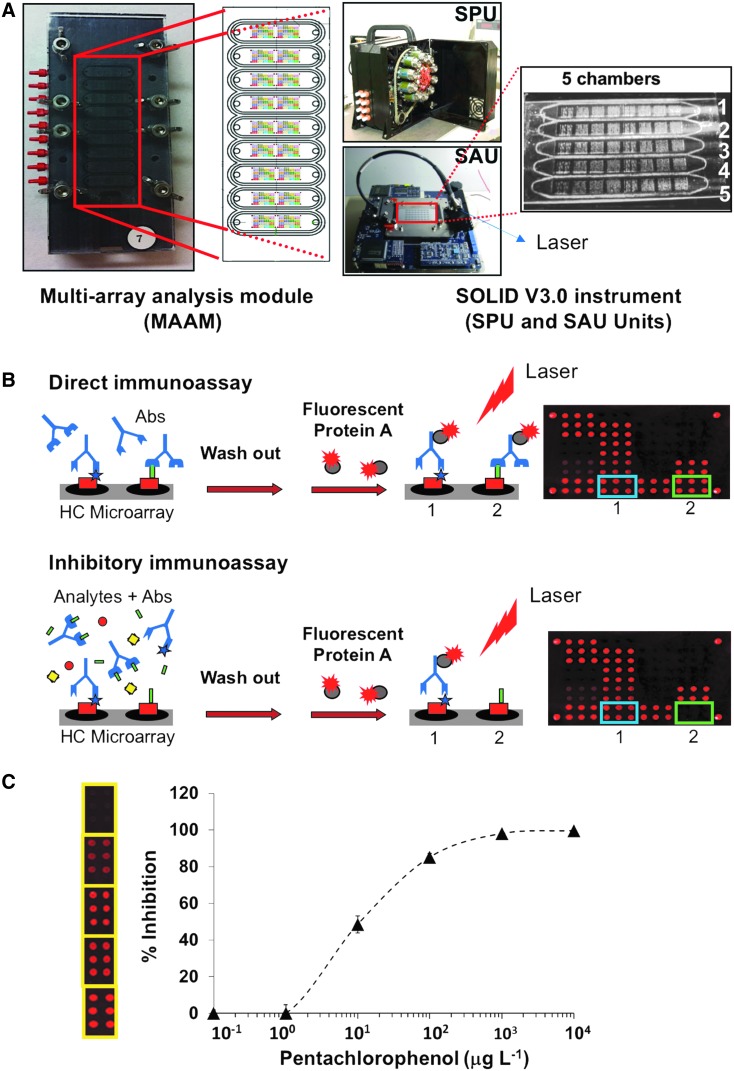
Multiplex inhibitory microarray immunoassays (MIMI). (**A)** MAAM device and SOLID instrument used for the inhibitory microarray immunoassays (Section 2). **(B)** Scheme showing how the inhibitory immunoassay works: Top: Antibodies are incubated with the hapten conjugate (HC) microarray without competitor/analyte as control for no inhibition. Antibodies (Abs) are captured by their corresponding printed hapten conjugates. The Abs not bound to conjugates are washed out, while those retained produce an image after incubation with Alexa 647-labeled protein A and laser-induced fluorescence excitation. The image represents 100% of FSI for each Ab-conjugate pair. Bottom: In a test sample, a mixture of antibodies is incubated with a liquid extract of natural soil sample so that the organic analytes present in the sample compete with the immobilized hapten conjugates on the microarray for binding to the antibodies. The analyte/antibody interaction is revealed as above. The competition of the analyte in the sample will reduce the fluorescence intensity of the corresponding spot in a proportional manner to its concentration. Observed signals are normalized to 100% by using the following formula: FSI = A/A_0_ × 100 where A_0_ is the fluorescence in the absence of analyte. **(C)** Calibration curves for each antibody were carried out. Example of a calibration curve of an inhibitory immunoassay for pentachlorophenol (right) and the corresponding microarray images used for quantification (left). FSI, fluorescence signal intensity; MAAM, multiarray analysis module; SAU, Sample Analysis Unit; SOLID, Signs of Life Detector; SPU, Sample Preparation Unit.

The targets were used either in their free, that is, unconjugated form, or linked to protein carriers such as keyhole limpet hemocyanin (KLH), bovine serum albumin (BSA), or ovalbumin (OVA). Protein conjugates of the targets were printed onto microscope slides following two patterns, one with nine identical microarray replicates to match with a multiarray analysis module (MAAM) cassette, and another one following the SOLID instrument pattern (Fig. 2). B[a]P was purchased from Sigma-Aldrich Co. (Madrid, Spain), and stock solutions at 1 mg mL^−1^ were prepared in acetone. D- and L-phenylalanine (D-Phe and L-Phe; >99% analytical degree) were purchased from Biochemika, Fluka (Sigma-Aldrich Co.). The free ModA peptide (MLAPLHKKIVYANTL) was produced by Sigma-Aldrich as described (Fernández-Calvo *et al.*, 2006). Standards of atrazine (ATZ) and pentachlorophenol (PCP) were purchased from Dr. Ehrenstorfer (Augsburg, Germany). Phthalylsulfathiazole (PSTZ), sulfamethazine (SMZ), and finasteride (FINA) were purchased from Fluka-Sigma-Aldrich Química (Madrid, Spain). Stock solutions of these analytes were prepared at 1 mg mL^−1^ in methanol and stored at −20°C until use. Working serial dilutions of each analyte (ranging in concentration from ppt to ppm) were prepared in phosphate-buffered saline solution (PBS; 10 mM phosphate, pH 7.4, 154 mM NaCl containing 0.01% BSA and 10% methanol). Alexa Fluor^®^ 647 protein A conjugate was obtained from Molecular Probes (Eugene, OR). Protein A stock solutions of 1 mg mL^−1^ were diluted 1:1000 in PBST buffer (10 mM phosphate, pH 7.4, 154 mM NaCl, 1% BSA, 0.01% Tween 20) for all immunoassays.

### 2.2. Microarray design and printing

Solutions of the protein-target conjugates were prepared in Whatman 1× protein arraying buffer (Sigma-Aldrich) containing 0.01% Tween 20, and printed onto epoxy-activated glass slides (Arrayit, Corporation) with a MicroGrid II TAS 600 spotting arrayer (BioRobotics, DIGILAB, Inc., Marlborough, MA). The conjugates of BSA and D- or L-Phe were used at 0.5 mg mL^−1^, while all other conjugates were used at 0.8 mg mL^−1^. BSA and PBS buffer were used as negative controls. The nine protein-target conjugates were spotted in sixfold replicates onto nine identical microarrays per slide. After spotting, slides were maintained for 10 min at room temperature (RT) and then stored at 4°C until needed.

For the inhibitory immunoassay, the slides were blocked by first immersing them in a solution of 0.5 M Tris–HCl, pH 9, containing 5% BSA, and then for 1 h under gentle agitation in 0.5 M Tris–HCl, pH 9, containing 2% BSA. Afterward, the slides were dried by short centrifugation and placed in a multiarray analysis cassette (MAAM) as previously described (Blanco *et al.*, 2016). The MAAM contains nine independent flow cells that match the nine arrays printed onto each slide. For SOLID instrument immunoassays (Parro *et al.*, 2011b), protein-target conjugates were printed in quintuplicate onto a specially designed 75 × 27 × 0.15 mm epoxy glass slide to obtain a regular pattern of five identical microarrays fitting into the five flow cells of the SOLID SAU.

### 2.3. Antibody titration

Direct microarray immunoassays were performed to determine the specificity, sensitivity, and optimal working concentrations for each analyte/antibody pair. Microarrays blotted with protein-target conjugates were blocked and set up in the MAAM as described above. Fifty microliters of a dilution series (1/500 to 1/64,000) of antibody in PBST was applied and incubated for 30 min at RT. One of the MAAM chambers containing only PBST buffer served as blank control. After a wash step with PBST buffer, 1 μg mL^−1^ of labeled Alexa-647 protein A in PBST, containing 1% BSA, was applied. After a 30-min incubation at ambient temperature in darkness, the slides were washed with PBST solution three times, dried, and scanned in a GenePix 4100A scanner (Molecular Devices, Sunnyvale, CA). Fluorescence intensities were plotted as a function of the logarithm of antibody dilution, and the optimal antibody dilution to be used in subsequent tests was determined as the middle point of the linear stretch where the regression value was higher than 98% (not shown).

### 2.4. Inhibitory microarray immunoassay

Each analyte/antibody pair was tested in an inhibitory setup to determine the limit of detection (LOD), as well as the inhibitor concentrations that reduce the maximum fluorescence signal intensity (FSI) by 50% (IC_50_), 75% (IC_75_), and 100% (IC_100_), respectively. Stock solutions of the free, unconjugated analytes were prepared in water at 1 mg mL^−1^, with the exception of ATZ, PSTZ, SMZ, PCP FINA, and B[a]P, which were dissolved in water:methanol (90:10). Tenfold serial dilutions of the stocks in PBST buffer yielded analyte concentrations ranging from ppm to ppb. Analytes were incubated with their corresponding antibody (at the optimal dilution) in 50 μL (total volume), at ambient temperature for 15 min with gentle mixing every 5 min. Then, each mixture was loaded into one of nine flow cells of the MAAM cassette and incubated for an additional 30 min. One flow cell was loaded with antibody in PBST buffer only, that is, without analyte, to determine the maximal FSI (100%). Following incubation, three washes with PBST were performed and Alexa-647 protein A was added. The slides were then washed three times by passing 1 mL of PBST buffer through the flow cells, dried by quick centrifugation, and scanned as described above. The fluorescence curves were fitted to a four-parameter logistic function according to the work of Brun *et al.* (2010).

### 2.5. Multiplex inhibitory microarray immunoassay

To assess the feasibility of analyzing mixtures of analytes and antibodies and detecting multiple analytes simultaneously, a multiplex inhibitory immunoassay was designed. For this purpose, a set of 10 antibodies and their corresponding targets were selected for MIMI (Table 1). Each antigen/antibody pair that had first been tested individually, as described above, was added one by one to the multiplex assay, thus increasing the total number of antigen/antibody pairs to 10. The assays were carried out in the MAAM cassette and the SOLID instrument (Parro *et al.*, 2011b). Printed slides were blocked as described above. For each multiplex assay, the previously determined optimal concentration of antibody was used, while the analytes were used at concentrations corresponding to their IC_50_ and IC_100_ values, respectively. The free analytes were first incubated together with the corresponding antibodies in 50 μL of PBST (total volume) for 15 min, before they were loaded into the MAAM chambers. After 30 min, the microarrays were washed with PBST buffer, incubated with Alexa 647-labeled protein A for another 30 min, washed, and scanned as described above.

Assays in the SOLID instrument were performed as follows: 0.5 g of kerogen-containing sample was loaded into one of the extraction cells and the organic material was extracted into 2.5 mL of double reinforced Tris buffered saline with Tween 20 (TBSTRR) aqueous buffer (0.4 M Tris–HCl pH 8, 0.3 M NaCl, 0.1% Tween 20) by ultrasonication. After filtering (5 μm cutoff), ca. 0.5 mL (total volume) of the liquid extract was mixed with antibodies, loaded into one of the five flow cells of the SOLID SAU, and incubated for 30 min with continuous flow for mixing. After washing with fresh buffer for 5 min, Alexa 647-labeled protein A was added. Following a 30-min incubation period and a washing step, the fluorescence image of the microarray was captured with the SOLID CCD camera after exciting the fluorochrome with a 635-nm excitation laser beam. One of the SAU chambers was filled with antibody-containing buffer only (i.e., in the absence of analyte) to obtain a 100% fluorescence intensity value. The presence of analytes in the sample liquid extract results in a reduction of the fluorescence intensity of corresponding spots that is proportional to the concentration in the sample; that is, the higher the concentration of the analyte, the lower the signal intensity in the microarray will be. An estimated error of about 5–10% was taken into account in the determination of the lower LOD of target compounds.

### 2.6. Image processing and data analysis

Slides were scanned on a GenePix 4100A scanner (Molecular Devices) or in the SOLID-SAU unit. Fluorescence intensities of the different spots on the microarray image were quantified with the GenePix Pro 6.0 software. Fluorescent signals were considered positive when they had an intensity of at least three times the background. The fluorescence intensities were plotted as a function of the antibody dilution to obtain a titration curve for each antibody. The optimal antibody dilution used in subsequent tests was determined as the dilution that resulted in 50% of the maximum FSI.

### 2.7. Analysis of natural soil samples

For an organic chemical analogue to the extreme martian environment, a natural soil sample from the Antarctic Dry Valleys (sample #726 provided by Dr. Chris McKay at NASA Ames Research Center, CA) was spiked with D-Phe, L-Phe, or a mixture of L-Phe and atrazine to perform three different experiments, as follows: (1) soil aliquots of 0.5 g were spiked with 0.5 mL of TBSTRR containing 1, 5, or 10 mg L^−1^ of D-Phe; (2) new 0.5 g soil aliquots were spiked with 20, 30, or 100 mg L^−1^ of L-Phe; and (3) a third set of samples spiked with the mixture of L-Phe at 20, 30, or 100 mg L^−1^, and atrazine at 10 μg L^−1^, 100 μg L^−1^, or 10 mg L^−1^, corresponding to the amounts that produced 50%, 75%, and 100% inhibition, respectively. After overnight incubation at 4°C, 2 mL of TBSTRR buffer was added to the mixtures and sonicated with a handheld sonicator (Hielscher 50W DRH-UP50H, Teltow, Germany) for 5 × 1-min cycles at maximum power (30 KHz) with fixed intervals of 2 min on ice. The mixtures were filtered with a 5 μm cutoff filter and analyzed in an inhibitory immunoassay using anti-D-AA (set 1), anti-L-AA (set 2), and a mixture of anti-L-AA and anti-atrazine antibodies (set 3).

Analytes and antibodies were allowed to interact in an Eppendorf tube for 15 min at ambient temperature before they were loaded into the MAAM and SOLID chambers and incubated for another 30 min with the conjugates on the microarray. After a washing step, detection was achieved by using Alexa fluor-647-labeled protein A and scanning of fluorescence as described above. As a 100% signal blank control, inhibitory immunoassays were carried out using the same antibody concentrations in the respective buffer without analytes. As a 100% inhibition control, an excess of analyte was used to obtain the maximal loss of fluorescence signal for each antibody.

### 2.8. Analysis of a type IV kerogen-containing sample

For organic chemical analogues to carbonaceous chondrites or degraded organic matter that may be encountered on Mars, we used aromatic compound-dominated type IV organic matter in oxidized “lignites” from Early Cretaceous fluvial sands and type IV organic matter from Late Jurassic paleosols collected in southern England; for a full description including detailed chemical analysis see Matthewman *et al.* (2013). Although very different in origin and history, type IV organic matter bears superficial organic chemical similarities to that found in carbonaceous chondrites and is useful for the purposes of instrument development and testing. The Early Cretaceous lignite (ECL) sample was analyzed in both the MAAM cassette (Section 2.4) and the SOLID instrument. First, the sample was washed to remove possible contamination that might have occurred during collection and shipment. To this purpose, 24 g of the sample was mixed with dichloromethane (DCM)/methanol (MeOH) 93:7 (v/v) for 2 min, followed by washing with ethanol, and drying at 70°C.

Next, the rock sample was crushed to a fine powder with a pestle and mortar, and an aliquot of 0.5 g was loaded into the extraction cell of the SOLID-SPU. Then, 2.5 mL of TBST_80_M buffer (Tris-HCl, 0.1 M, pH 8, 0.15 M NaCl, 1.5 g L^−1^Tween 80, and 20% methanol final; Sims *et al.*, 2012) was pumped into the SPU extraction cell, and five cycles of ultrasonication were applied with 2–3-min intervals, reaching a pressure of 2.7 bar and a temperature of up to 80°C. Afterward, the mixture was filtered through a 10-μm filter to remove any large particulate matter. The filtrate constituted the crude extract that was analyzed in the MAAM cassette or in the SOLID-SAU. A mixture of six antibodies (anti-B[a]P-4F11, anti-B[a]P-5G1, anti-phthalylsulfathiazole, anti-ModA peptide, anti-D-AA, and anti-L-AA 18.3) was incubated for 15 min at RT in the microarray-containing MAAM cassette, and processed as described above. For analysis in the SOLID instrument, the sonicated and filtered crude extract was pumped from the SPU to one of five microarray flow cells in the SAU. After 30 min of incubation, the microarray was washed with TBSTRR buffer for 5 minutes, before fluorescence was excited by a laser beam and imaged with the CCD camera.

### 2.9. Analysis of the ECL sample by gas chromatography/mass spectrometry

To validate the MIMI, the B[a]P content in the kerogen sample (ECL) was also determined by gas chromatography/mass spectrometry (GC-MS). Two grams of the ECL sample was extracted for 2 h with acetone using a Büchi model B-811 automatic extractor. The organic extract was concentrated to 500 μL by heating and then analyzed directly by GC-MS with an Agilent 6850 GC system coupled to an Agilent 5975 VL MSD with triple-axis detector operating in electronic impact mode at 70 eV. The GC column was an HP-5MS column coated with (5%-phenyl)-methylpolysiloxane. The operation conditions were as follows: 1.1 mL/min He carrier gas; initial temperature hold at 60°C for 3 min; increase from 60°C to 230°C at a rate of 15°C/min; hold 5 min; increase from 230°C to 300°C at a rate of 10°C/min; and hold for 5 min. The sample was injected in splitless mode with the injector temperature at 325°C. The temperature of the ion source was 250°C, and the quadrupole temperature was 180°C. Data were acquired and processed with the Agilent Chemstation software. An external standard of B[a]P purchased from Sigma-Aldrich was used for the identification of the peak attributed to B[a]P based on retention time and mass spectrum. Quantification of the analyte was achieved by using the multiple point external standard method.

## 3. Results

### 3.1. Highly sensitive multiplex inhibitory immunoassays for detecting aromatic compounds in environmental samples

Amino acids and aromatic compounds of diverse structures are relevant targets as biomarkers in the search for extraterrestrial organic matter, while the type IV organic matter-rich samples represent good terrestrial analogues for the development of new and sensitive methods suitable for planetary exploration. Here, nine aromatic compounds (Fig. 1), including simple organic molecules, environmental contaminants, synthetic compounds, and potential molecular biomarkers, (Table 1) were selected as targets for the development of a sensitive MIMI.

**FIG. 3.**
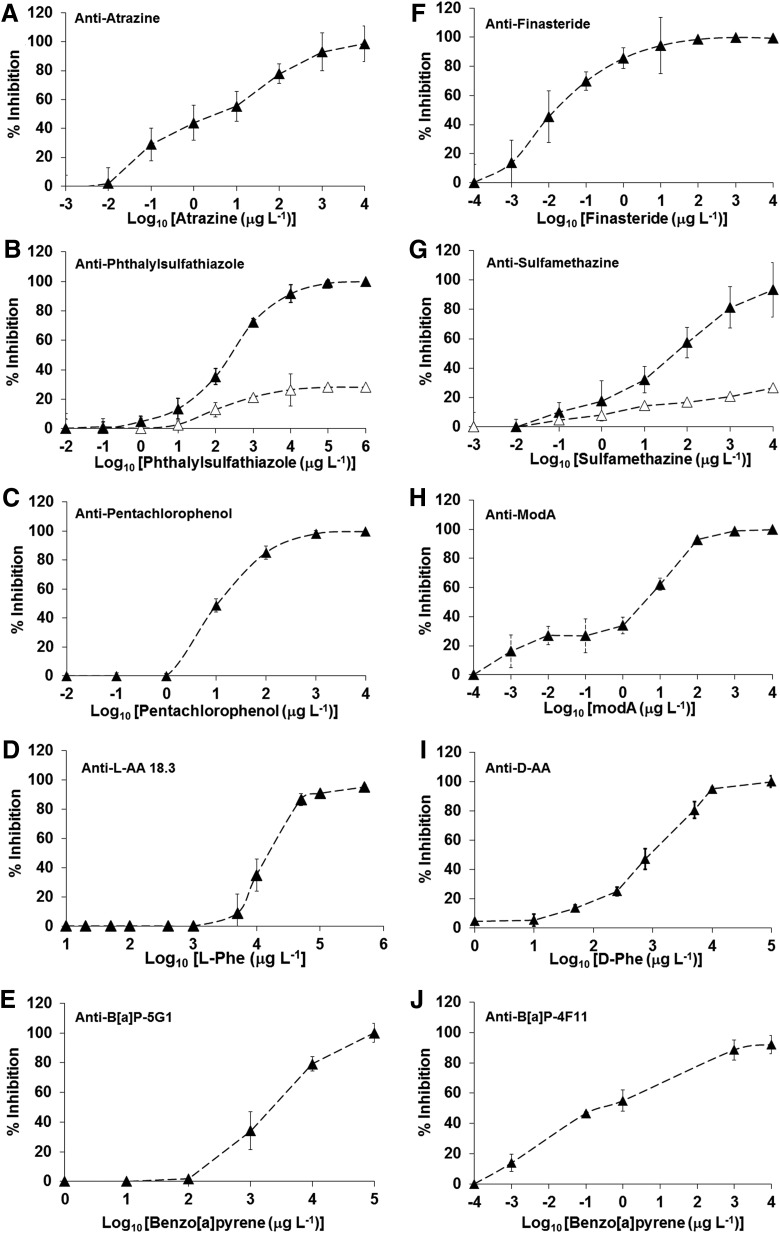
Standard calibration curves obtained by single inhibitory immunoassay for each analyte/antibody pair. **(A–J)** Calibration curves showing the base 10 logarithms of the concentration of free analytes in solution versus the percentage of inhibition (% Inhibition). Percentage of inhibition was calculated by using the following formula: (1 − A/A_0_) × 100; where A_0_ and A are the fluorescence signals observed in the absence and presence of the inhibitor, respectively. Results represent averages of six tests performed on each of three different arrays. Error bars represent the standard deviation of three experiments. Sigmoidal curves have been fitted to a four-parameter logistic function for determining the IC_50_ (Table 1). Serial dilutions of analytes were diluted in PBST buffer (Tween 20, 0.01%) with the exception of B[a]P, which was diluted in PBST and 10% methanol (see Section 2 for details). In **(B, G)**, filled triangles (▴) indicate the percentage of inhibition corresponding to the analyte phthalylsulfathiazole, and open triangles (▵) indicate the percentage of inhibition obtained for the analyte sulfamethazine for both, anti-phthalylsulfathiazole and anti-sulfamethazine antibodies.

First, the specificity of all antibodies was tested in a direct, noninhibitory fluorescence immunoassay by applying each antibody individually to the microarray comprising the whole set of 10 protein/analyte conjugates (Fig. 2B). This setup allowed an assessment of the potential cross-reactivity of any of the antibodies with spots other than those containing the corresponding target. While with most antibodies no cross-reactivity was observed, a low signal (<5%) was obtained with anti-D-AA on the L-Phe-conjugate when antibody concentrations of equal to, or higher than, 2 μg mL^−1^ were used. In the case of the anti-phthalylsulfathiazole and anti-sulfamethazine antibodies, they showed consistent and reproducible cross-reactivity in both the direct (not shown) and the inhibitory immunoassay (Fig. 3). Calibration curves for each antibody/analyte pair were determined, and the optimal antibody dilution for all subsequent immunoassays was inferred from the linear part of the curves (not shown). We determined the LOD for all analytes, as well as the corresponding IC_50_ and IC_100_ values (Table 1). Sensitivities ranged from ppb for most analytes to ppt (B[a]P with anti-B[a]P-4F11, finasteride, atrazine, and ModA).

Our results confirm LOD values similar to those reported by Kassa *et al.* (2011) with another microarray format for anti-L-AA and anti-D-AA antibodies (Fig. 3), although we detected nearly 5% of cross-reactivity with L-Phe conjugate when using the anti-D-AA polyclonal (as indicated above). No cross-reactions were found for both anti-D- and anti-L-AA antibodies when L- and D-enantiomers of the free amino acid phenylalanine were, respectively, assayed in inhibitory assays. In addition, anti-L-AA and anti-D-AA were able to recognize other aromatic amino acids such as Trp and Tyr (few ppm) in separate assays, but neither recognized the aliphatic amino acid alanine at the concentrations used, nor glycine (not shown).

**FIG. 4.**
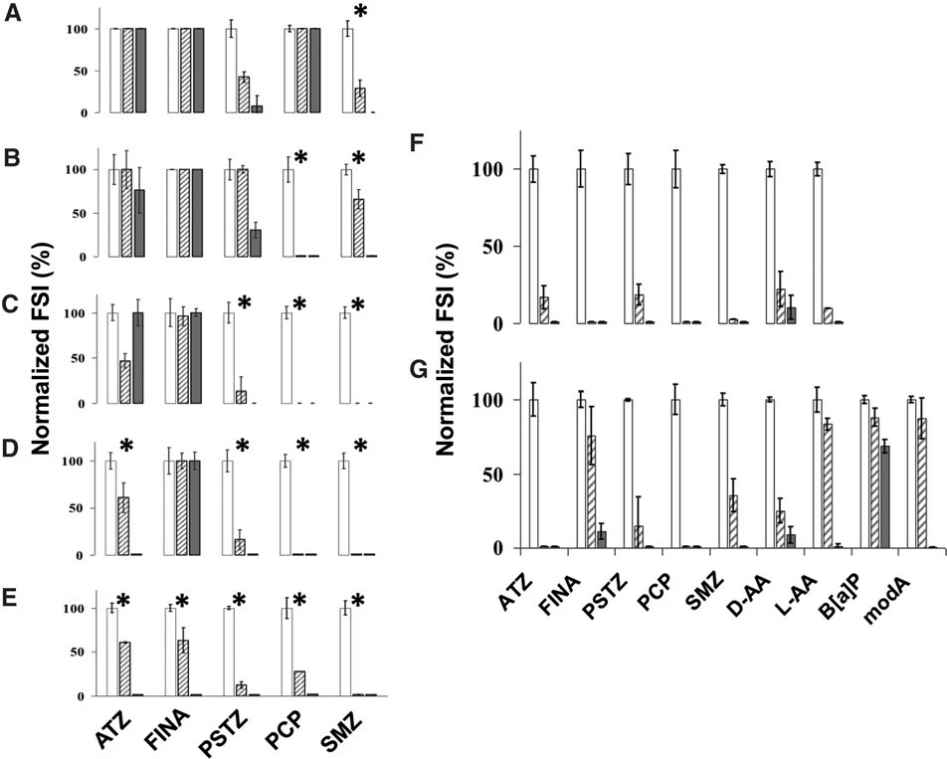
Multiplex inhibitory microarray immunoassays (MIMI). **(A)** The fluorescence obtained in each hapten conjugate spot after incubation with the 5-antibody mixture, without inhibitors, was quantified and normalized as 100% fluorescence (white bars, no inhibition). **(A–E)** Sequential and specific antibody binding inhibition by using the mixture of five antibodies and their corresponding inhibitors/analytes added in a stepwise manner as indicated by asterisks (*). Inhibitors/analytes were added in two different concentrations to account for each IC_50_ (hatched bars) and IC_100_ (gray bars). **(F, G)** MIMI using seven and nine antibodies, respectively. ATZ, atrazine; PCP, pentachlorophenol; PSTZ, phthalylsulfathiazole; SMZ, sulfamethazine; FINA, finasteride; D-aa, D-Phe; L-aa, L-Phe; B[a]P, Benzo[a]pyrene; and ModA peptide.

An MIMI was then developed to enable simultaneous analysis of the analytes. To assess potential interactions between different antibodies and analytes, inhibitory multiplex immunoassays were performed by adding the analytes in a stepwise process to the same antibody mixture (Fig. 4). The inhibition data obtained with the increasing analyte mixtures (Fig. 4A–E) indicated that some cross-reactivities and/or interferences were occurring. For example, the aforementioned reproducible interaction between the anti-phthalylsulfathiazole and anti-sulfamethazine antibodies, and their targets, which may be due to structural similarities of the sulfonamide group shared by both compounds (Fig. 4A), was confirmed. Also, the addition of phthalylsulfathiazole seemed to affect the inhibition of anti-atrazine at the IC_50_ dose. However, the fact that this effect was not observed when higher doses (IC_100_) were used (Fig. 4C), and that expected responses were obtained after the addition of atrazine (Fig. 4D), suggested that this was an experimental artifact.

The inhibition effect increased with mixtures of seven antibodies at IC_50_ inhibition concentrations as determined individually with each analyte/antibody pair (Fig. 4F). Even in some cases no significant differences were observed in the inhibition between the IC_50_ and IC_100_ amounts (Fig. 4). It seemed that the higher the number of antibodies used in the mixtures, the higher the inhibition effect obtained in most of them. These results confirm the vulnerability of multiplex inhibitory assays, where the cross-reactivity increases with the number of targets, as was reported by others (Ellington *et al.*, 2010). Consequently, multiplex inhibitory immunoassays for the detection of small molecules are usually reduced to a few targets (Szkola *et al.*, 2014; Carter *et al.*, 2016). Thanks to the microfluidics and micro- and nanoprinting techniques, this drawback can be overcome by running several multiplex inhibitory immunoassays in microfluidic devices (Cao *et al.*, 2015).

**FIG. 5.**
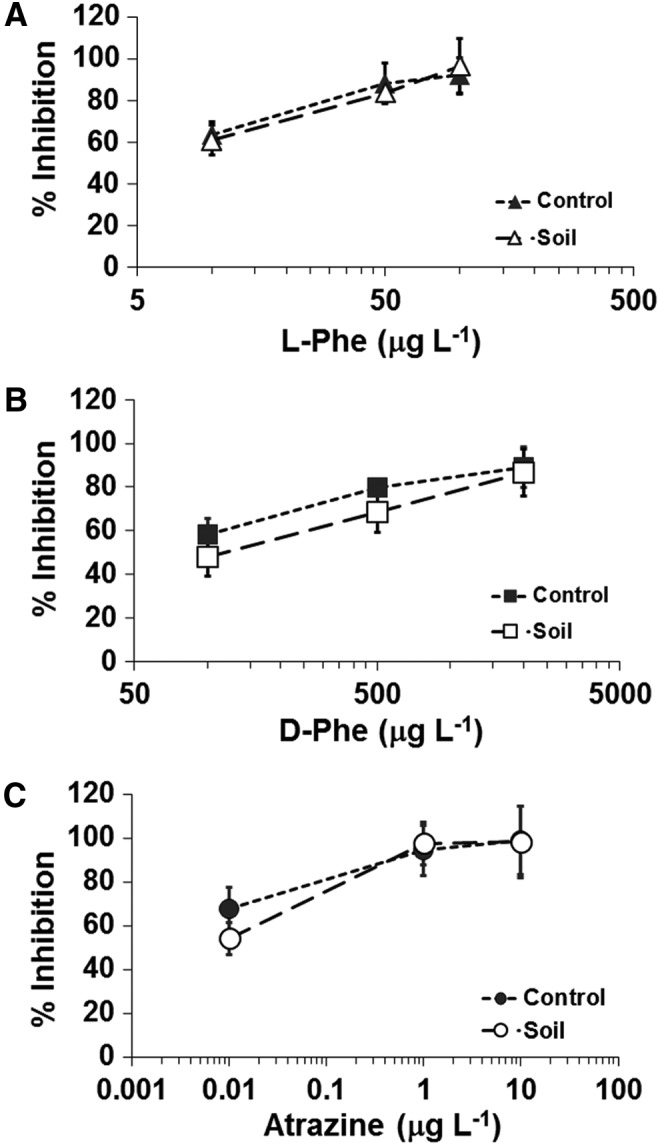
Detecting organics and aromatic amino acids with chiral selectivity by inhibition immunoassays in spiked soil samples. Antarctic soil samples were doped with IC_50_, IC_75_, and IC_100_ concentrations (open symbols) of free L-Phe **(A)**, D-Phe **(B)**, and atrazine **(C)**, extracted, and analyzed by inhibition immunoassay with the corresponding antibody. Parallel assays with the same compounds and concentration were performed as controls without soil extract (closed symbols).

To test the effect that minerals or other complex compounds contained in natural samples might have on the inhibitory immunoassay, we selected a soil sample from the Antarctic Dry Valleys because of its low biomass content and, potentially, absence of drug and pesticide contaminants. The sample was spiked with D-Phe, L-Phe, and a mixture of L-Phe and atrazine at concentrations corresponding to their respective previously estimated IC_50_, IC_75_, and IC_100_. A comparison of the resulting fluorescence intensities with values obtained in the absence of soil (Fig. 5) showed no significant effect of the soil sample. While the results obtained with D-Phe at 100–500 ppb in the presence of soil are overall lower than those obtained in the absence of soil, the deviations are within the experimental error of the method.

### 3.2. Detection of B[a]P in type IV kerogen by a multiplex inhibitory immunoassay

**FIG. 6.**
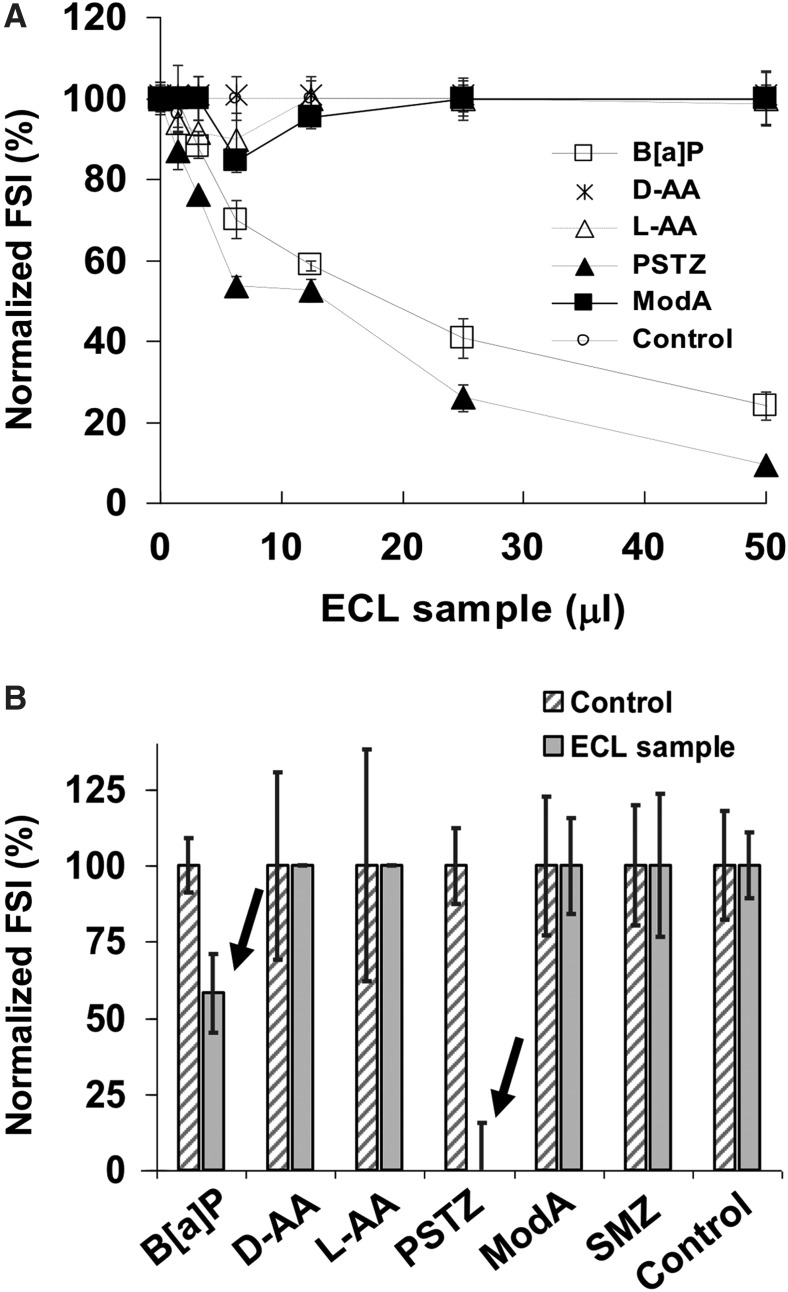
MIMI for the detection of small organic compounds by using both MAAM and SOLID devices. **(A)** Normalized fluorescence intensity of a series of twofold dilutions of an ECL extract assayed in the MAAM device. A mixture of six fluorescent antibodies (anti-sulfamethazine, anti-phthalylsulfathiazole, anti-L-AA 18.3, anti-D-AA, anti-ModA peptide, and anti-B[a]P-5G1) at their optimal concentration was mixed with different amounts of the ECL extract and assayed for inhibition of binding to the hapten conjugate microarray (Section 2). The results are the average of two replicate experiments. **(B)** The plot displays the MIMI performed with 50 μl of ECL extract in SOLID instrument by using a mixture of six antibodies indicated above (see Section 2). Hatched bars, no sample controls (100% of FSI); gray bars, loss of signal after MIMI with ECL extract. Arrows show inhibition effects with B[a]P and phthalylsulfathiazole of about 50% and 100%, respectively.

The sample from the ECL was used to assess the suitability of the multiplex inhibitory immunoassay for the detection of small organic compounds such as the PAH B[a]P and aromatic amino acids. Lignites are sedimentary rocks formed from post-burial processes with a carbon content of around 60–70% containing PAHs. Six antibodies (anti-B[a]P-4F11, anti-B[a]P-5G1, anti-Phthalylsulfathiazole, anti-ModA-peptide, anti-L-AA 18.3, and anti-D-AA) were mixed with serial dilutions of a rock sample extract and tested by MIMI with several amounts of ECL liquid extract, both by manual operation in the MAAM device and semiautomatically in the SOLID instrument (see Section 2). The results show a strong decrease in fluorescence intensity in those spots corresponding to B[a]P and PSTZ conjugates (Fig. 6), indicating that phthalylsulfathiazole and B[a]P were detected both by the MAAM (Fig. 6A) and by the SOLID instrument (Fig. 6B) using as little as 2.5 μL and 5 μL of the sample extract, respectively.

Based on the calibration curves obtained for B[a]P with 4F11 and 5G1 antibodies, we estimated that the ECL sample contained B[a]P at a concentration between 2 and 6 ppm. The concentration of phthalylsulfathiazole-like compounds in the ECL was estimated between 5 and 6.4 ppm. In addition, significant and reproducible reductions in signal intensities (by nearly 15%) were also obtained with two other antibodies, anti-L-AA and anti-ModA peptide (directed against a peptide conjugate), suggesting the presence of aromatic L-amino acids and possibly some cross-reacting peptides.

**FIG. 7.**
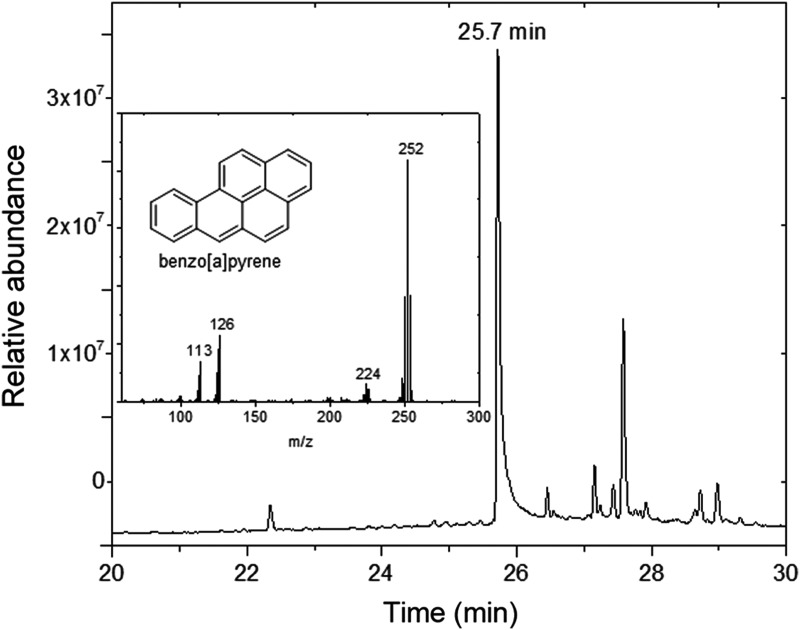
Detection of the B[a]P in the ECL sample by GC-MS analysis. The retention time at 25.7 min in the chromatogram and the mass spectrum (insert) are indicated.

To further validate the immunological results, we carried out organic extraction and GC-MS analysis to identify and quantify B[a]P in the ECL sample (Fig. 7). The measured concentration was about 44 ppm, which was nearly 10 times more than the one determined with the immunoassay. This discrepancy might be due to different extraction efficiencies rather than the analytical technique itself, because each method uses its own buffer for sample processing and extraction (see Sections 2 and 4).

## 4. Discussion

### 4.1. Sensitive MIMI for the detection of aromatic organics in a type IV kerogen analogue

The use of anti-L-AA and anti-D-AA antibodies has been previously reported for the detection of small molecules that only differ in their stereochemistry or a functional group by multiplex fluorescence microarray immunoassays (Kassa *et al.*, 2011). Herein, we have increased the number of antibodies, expanded the versatility, and demonstrated the usefulness of MIMI assays for simultaneous stereoselective recognition and identification of universal chemical structures such as aromatic amino acids and PAHs, and other compounds (Fig. 4). In addition, we have validated the assay through a semiautomatic process with the SOLID instrument by detecting PAHs and sulfur-containing aromatics in a highly mature kerogen sample (Fig. 6).

No significant interferences were recorded in the MIMI assays using extracts from an Antarctic soil sample, and the inhibition value was similar to the control that did not contain soil (Fig. 5). In the case of anti-D-AA, the effect of soil seemed to impair the inhibition effect of D-Phe by about 10% in comparison to values obtained for the control. Considering that anti-D-AA and anti-L-AA antibodies can detect the three aromatic amino acids and that no significant alteration of the inhibition curves was observed with the spiked soils, the results suggest the absence of amino acids in this sample as previously concluded by others (Brinton *et al.*, 1998).

The Murchison meteorite is dominated by aromatic structures that reside mainly, but not exclusively, in a high-molecular-weight organic network (Sephton and Gilmour, 2000). The meteoritic aromatic network is relatively robust and persists in meteorites despite the level of secondary thermal or aqueous processing that has occurred on their parent bodies (Sephton *et al.*, 2003). The widespread nature of the meteoritic aromatic network makes it a sensible representative of solar system abiotic organic matter. The analysis of the type IV organic matter-containing samples by MIMI assay showed the presence of B[a]P and sulfonamide-like compounds. The presence of B[a]P as well as sulfur-containing aromatics in kerogens is well documented as a consequence of diagenetic processes operating on biological matter (Rullkötter and Michaelis, 1990; Killops and Killops, 2005). However, sulfonamides are antibiotics of broad spectrum used in medicine and as feed additives (Zhang and Wang, 2009; Wang *et al.*, 2013). Polyclonal antibodies to sulfonamides have been successfully applied for the detection of these compounds at ppb level in the environment (Pastor-Navarro *et al.*, 2007).

The detection of a phthalylsulfathiazole-like compound in lignite samples might indicate the presence of organic sulfur compounds (OSC) that could share a chemical structure similar to the sulfone group. It has been reported that fossil fuels that derive from ancient organisms, such as coal and petroleum, often contain OSC (Gryglewicz and Rutkowsk, 2001). Pristine coals contain sulfur in a reduced state, such as sulfoxides, sulfones, sulfonic acid, and sulfates. Lignites are commonly associated with iron sulfates such as jarosite (M. Sephton, personal communication). In addition, Fang-Jing *et al.* (2015) reported the presence of the sulfone group in 44.5% of all sulfur forms in lignite stemming from the Xianfeng coal mine. Sulfones are considered unreactive because of the rigidity of the functional group (R-S( = O)_2_-R′), which explains its persistence in coals and other materials for long periods of time. The absence of sulfamethazine cross-reactivity in the ECL sample, which is always detected along with the phthalylsulfathiazole in MIMI assays, might indicate the presence of thiazole groups, which are also formed in OSC as part of the diagenesis processes (Noriyuki and Philp, 1995).

While we used acetone as a solvent for exhaustive thermal extraction by Soxhlet for GC-MS analysis, the extraction for MIMI was based on an aqueous formulation (see Section 2) and limited ultrasonication. The MIMI extraction buffer contained 20% methanol (Court *et al.*, 2012) to help extracting some organic compounds, but we could not increase this concentration further because the buffer is also used as an incubation buffer for the immunoassay (to save mass and gain simplicity in planetary exploration instruments). Therefore, a lower efficiency in the extraction by the aqueous solvent used in the SOLID instrument may account for the 10 times difference in the B[a]P concentration estimation. Further work is required for improving and optimizing the extraction by sonication with the SOLID system.

### 4.2. MIMI for detecting complex organics in planetary exploration

The antibodies selected for this study all bind to compounds that are highly relevant for the search of complex organics and molecular evidence of life in planetary exploration. The detection of aromatic amino acids, in particular their D- and L-enantiomers, may provide strong evidence, although not definitive proof, of life. The anti-L-AA and anti D-AA antibodies used here are stereoselective and, at the same time, versatile enough to detect the general amino acid functional grouping of structurally different amino acids having the correct configuration (Hofstetter *et al.*, 1998, 2000). Similarly, the fact that the anti-phthalylsulfathiazole antibody allowed us to detect thiazole-like groups in the kerogen sample demonstrates that antibodies can have relaxed specificities, and can be used for analyzing molecules with similar three-dimensional structures. This is especially relevant in the search for life in planetary exploration, where extraterrestrial life forms may not use exactly the same molecular structures as those on Earth. Searching for life elsewhere is always based on preconceived ideas developed by way of our terrestrial experiences.

Even if we search for a “second genesis” of a completely different life, we search for molecular structures, or patterns, that somehow allow us to compare with life as we know it. Although bioaffinity systems such as the immunoassays might be apparently too specific for a search for unknown molecular biomarkers in planetary exploration, antibodies and other similar molecular binders (lectins, aptamers, and molecularly imprinted polymers) can be designed and produced to recognize and bind to universal chemical forms. This is the case for some of the antibodies shown herein like the anti-L-AA 18.3 and anti-D-AA, which have the capability to bind the general amino acid functional grouping. Implementing MIMI for planetary exploration is challenging, but we suggest that, by selecting a few tens of universal molecular structures and producing the corresponding molecular binders (e.g., antibodies or aptamers), it can contribute significantly in the search for evidence of life in the exploration of Mars or the ocean worlds of Jupiter and Saturn.

We validated the MIMI assay by using six antibodies and the hapten conjugate microarray to search for aromatic compounds relevant for planetary exploration in kerogens. PAHs are ubiquitous in space and carbonaceous chondrites and are common environmental pollutants (Pizzarello and shock, 2010; Karsunke *et al.*, 2011). The detection of B[a]P by using two different and redundant monoclonal antibodies with amounts of sample extract as little as 3 μL validated the MIMI assay (Fig. 6). These results were in agreement with those of Matthewman *et al.* (2012), who reported recognizable PAHs in a fossil forest GDB sample. The discrepancy between the concentration of B[a]P in the kerogen determined with MIMI and GC-MS might be explained by differences in the efficiency of the extraction methods.

Space exploration constraints, such as mass, volume, energy, or redundant mechanisms, require the simplifying of procedures and the analytical techniques. For the sake of such simplicity, we use a single water-based liquid formulation with surfactants for both the organic extraction (hydrophobic and hydrophilic compounds) and the immunoassay (Court *et al.*, 2012). Such a buffer has to be, at the same time, mild enough to leave intact the antibody performance, which implies that significant sacrifices and trade-offs are required to accommodate the protein-based immunoassay detector. Such trade-offs mainly affect the most hydrophobic fraction of the organics, which is usually covered, although with their own constraints, by other techniques such as the organic solvent extraction and GC/MS analysis performed by the MSL SAM instrument (Freissinet *et al.*, 2015). However, our water-based extraction by ultrasonication has the advantage, besides its simplicity, of the molecules remaining intact and unaltered by high temperature (>300°C), and their chemical information complete (Parro *et al.*, 2011b).

We have proposed the SOLID concept as payload for planetary exploration missions in several calls for instruments such as ESA's ExoMars, NASA's MSL, and Mars2020, or NASA's Discovery 2014 as part of the Icebreaker mission proposal (McKay *et al.*, 2013). Important mission constraints have been addressed in all cases, such as the resistance of antibodies or other reagents to space conditions during the mission, planetary protection issues, contamination controls, and potential false positive results and how to manage them. Along with other authors, we have reported that antibodies and other biochemical materials have the capability to resist certain conditions with regard to space and other high stress environments such as high vacuum, thermal cycling (de Diego-Castilla *et al.*, 2011), and ionizing radiation (Thompson *et al.*, 2006; Baqué *et al.*, 2011; de Diego-Castilla *et al.*, 2011; Derveni *et al.*, 2012; Carr *et al.*, 2013), and it is estimated that such biochemical materials would remain viable over the course of, for example, a 2-year mission to Mars.

Planetary protection procedures are put in place to avoid (1) forward contamination of terrestrial life and (2) the introduction of organic/biochemical materials that could be confused with life detection target molecules. For the former, the proposed sterilization method for SOLID is to assemble individually treated and sterilized components in an aseptic environment. The reagents and bioaffinity sensors (antibodies, aptamers, lectins, or others) are also sterilized separately (filtration). For the latter, all reagents will always be confined in hermetic compartments and isolated by a biobarrier from other compartments and instruments. Moreover, the SOLID antibody detectors are extremely well characterized and should not be confused with any target materials when coupled with organic contamination recognition.

Organic contamination information will be provided by procedural blanks on Earth using preflight systems. Also, procedural blanks, on arrival at an extraterrestrial destination and before operation at the surface, will assess any damage or disruption to the antibody detectors. These tests will also provide a baseline assessment and potential false positive identification. Similarly, internal positive controls of a sterilized sample spiked with a well-characterized compound, preferably a human-made substance highly improbable to be encountered beyond the confines of Earth, will prove the well-functioning of the system.

## 5. Conclusions

An MIMI has been developed for the detection of organic compounds in soils and extraterrestrial sample analogues. Type IV kerogen from geological horizons on Earth bears superficial chemical similarities to the Murchison organic matter (Matthewman *et al.*, 2013) that makes it a good analogue for testing analytical techniques and instruments designed to discriminate between abiotic and biotic organic matter outside Earth. Mixtures of antibodies against small molecules can be used to search for a variety of compounds, including aromatic amino acids, PAHs, or thiazole-like compounds in organic chemical analogues of carbonaceous chondrites. The system was implemented into the SOLID instrument and designed and dedicated for the search for molecular evidence of life in planetary exploration.

We have demonstrated the feasibility of a mild water-based extraction system and the multiplex immunoassay for detecting life- and nonlife-derived organics with the high technological readiness level (TRL) SOLID prototype. Additional efforts, however, will need to focus on the improvement of solvent composition and extraction efficiency, and at the same time maintain compatibility with the immunoassay (or other biosensing assays) and the simplicity of the procedures. Our data demonstrate the ability and versatility of the MIMI system for detecting biological and nonbiological organic compounds in mineral matrices in a high TRL instrument for *in situ* analysis. The concept is particularly applicable to Mars and other life-search targets as well, such as the water-rich worlds of the outer Solar System.

## Notes

Associate Editor: Christopher McKay

## Abbreviations Used

B[a]P: benzo[a]pyrene
BSA: bovine serum albumin
ECL: Early Cretaceous lignite
FSI: fluorescence signal intensity
GC-MS: gas chromatography/mass spectrometry
KLH: keyhole limpet hemocyanin
LOD: limit of detection
MAAM: multiarray analysis module
MIMI: multiplex inhibitory microarray immunoassays
MSL: Mars Science Laboratory
OSC: organic sulfur compounds
OVA: ovalbumin
PAHs: polycyclic aromatic hydrocarbons
PBS: phosphate-buffered saline solution
SAM: Sample Analysis at Mars
SAU: Sample Analysis Unit
SOLID: Signs of Life Detector
SPU: Sample Preparation Unit
